# Refractory Seizure in a Patient With Griscelli Syndrome: A Unique Case With One Mutation and a Novel Deletion

**DOI:** 10.7759/cureus.14402

**Published:** 2021-04-10

**Authors:** Juan Fernando Ortiz, Samir Ruxmohan, Ivan Mateo Alzamora, Amrapali Patel, Ahmed Eissa-Garcés

**Affiliations:** 1 Neurology, Universidad San Francisco de Quito, Quito, ECU; 2 Neurology, Larkin Community Hospital, Miami, USA; 3 Neurology, Larkin Community Hospital, Miami, Florida, USA; 4 Medicine, Universidad San Francisco de Quito, Quito, ECU; 5 Public Health, George Washington University, Washington, USA

**Keywords:** seizures, griscelli syndrome

## Abstract

Griscelli syndrome (GS) is a rare syndrome characterized by hypopigmentation, immunodeficiency, and neurological features. The genes Ras-related protein (RAB27A) and Myosin-Va (MYO5A) are involved in this condition's pathogenesis.

We present a GS type 1 (GS1) case with developmental delay, hypotonia, and refractory seizures despite multiple medications, which included clobazam, cannabinol, zonisamide, and a ketogenic diet. Lacosamide and levetiracetam were added to the treatment regimen, which decreased the seizures' frequency from 10 per day to five per day. The patient had an MYO5A mutation and, remarkably, a deletion on 18p11.32p11.31. The deletion was previously reported in a patient with refractory seizures and developmental delay. We reviewed all cases of GS that presented with seizures. We reviewed other cases of GS and seizures described in the literature and explored possible seizure mechanisms in GS. Seizure in GS1 seems to be related directly to the MYO5A mutation.

The neurological manifestations in GS2 seem to be caused indirectly by the accelerated phase of Hemophagocytic syndrome (HPS), which is characteristic of GS2. By having the MYO5A gene mutation combined with the 18p11.32p11.31 deletion, the prognosis and severity of the patient's condition are poor. This is the first report of GS1 with a deletion in 18p11.32p11.31.

## Introduction

Griscelli syndrome (GS) is an autosomal recessive disorder characterized by hypopigmentation, immunodeficiency, and neurological features [1]. The syndrome was first described in 1978 [2]. Only 150 cases have been reported to date, with a higher prevalence in Mediterranean countries such as Turkey [2-3]. GS usually manifests from four months to four years, but other studies have reported a range from one month to eight years [2].

Two genes on chromosome 15q21, Ras-related protein Rab-27A (RAB27A) and Myosin-Va (MYO5A), are the cause of GS [1]. GS type 1 (GS1) presents with partial albinism and neurological features while GS type 2 (GS2) presents with partial albinism and immunodeficiency [4]. GS type 3 (GS3) is caused by a mutation in chromosome 2q37.1, which encodes melanophilin; the disease is usually benign [1]. RAB27A has an immunologic effect. The gene is expressed in cytotoxic T cells and natural killer cells [4]. The gene regulates the docking of proteins and exocytosis of granules containing granzyme and perforins. The dysfunction in cytotoxic T cells and natural killer cells explains the immunodeficiency seen in GS2. The gene MYO5A regulates the organelle transport in melanocytes and neuronal cells [5].

GS is an extremely rare disorder. Knowing the peculiarities of the clinical presentations is difficult because there is not sufficient information. We present a case of GS1 with an additional deletion (18p11.32p11.31). This deletion was previously reported by Verroti et al. [6]. This patient presented with similar clinical features as our patient, including drug-resistant atypical absence epilepsy and severe developmental delay [6]. The main concern of the patient was refractory seizures. We have presented the differential diagnosis of the disorder, differentiated each type of GS, and reviewed cases of GS with seizures. Finally, we have discussed the possible causes of seizures and the prognosis in each type of GS.

## Case presentation

History of present illness

A three-year-old male was brought to the hospital by his mother due to increased seizure frequency despite being on multiple anti-seizure medications. The patient was admitted for further assessment. Past medical history was relevant for epilepsy, infantile spasms, developmental delay, gastroesophageal reflux, laryngomalacia, and dysphagia. The mother denied any prenatal or neonatal complications. A genetic test showed a mutation in the MYO5A gene of chromosome 15q21, suggesting GS.

Seizure history

The child was diagnosed with infantile spasms at 11 months of age, and he was successfully treated with prednisone. He was started on clobazam for seizure prophylaxis, but he developed myoclonic seizures after two months. Clobazam dose was adjusted, which helped reduce the frequency of the seizures.

At 16 months of age, he started having tonic-clonic seizures and was started on a ketogenic diet, which decreased the seizures' frequency from 20 episodes to 10 episodes a day. He was also given cannabinol at age two, which decreased the seizures' frequency to less than five per day. However, seizures recurred again with frequencies above 10 per day.

At two years and three months, he was started on levetiracetam without any significant change. Three months later, zonisamide was given without any significant effects. The child's neurologist felt that the ketogenic diet was no longer helpful, and, therefore, the patient was weaned off the diet. The dose of zonisamide was increased, which reduced the seizures' frequency to four to five episodes a day.

Physical exam

Table 1 shows the results of the physical exam of the patient.

**Table 1:** Physical exam of the patient HEENT: head, eyes, ears, nose, and throat; Kg: kilograms

Physical Exam			
Weight	13.1 kg	HEENT	Hypopigmented eyebrows, eyebrows, light blonde hair, and a small nose
Cardiopulmonary	No extremity edema, murmurs, or pathological pulmonary sounds.	Gastrointestinal-renal	No abnormalities
Skin	No visible or palpable lesions. There are no cutaneous stigmata of neurological disease.	Psychiatric	Global delay
Neurological Exam			
Cranial nerves	Intermittent disconjugate eye movements, face symmetric, maintains head when turned to the right	Motor	Generalized axial and appendicular hypotonia, the head is lagging. No tremors or dyskinesia seen; the muscle bulk is decreased
Sensory	Normal sensation	Gait and Reflexes	Unable to maintain sitting position, marked head lag, 2+ biceps, 1+ patella bilaterally. No clonus. Toes going down.
Developmental History			
Gross motor	It was delayed prior to spasms at age 11 months, he can roll over, he has difficulty in maintaining a sitting position	Speech	Babbles, no words.
Social	Does not interact with siblings. No separation anxiety. Cries when he wants something.	Fine motor	Pincer grasp

Diagnostic assessment

Laboratory

White blood cell (WBC) count was 5.8 × 109/L, hemoglobin (Hb) 13.1 g/dl, hematocrit (HCT) 36.2%, platelet (PLT) 161 × 109/L, Na 137 mEq/L, K 4.5 mEq/L, CO_2_ 20.0 mEq/L, Cl 101 mEq/L, Cr 0.18 mg/dL, and blood urea nirogen (BUN) 4 mg/dL.

Magnetic Resonance Imaging (MRI)

**Figure 1:**
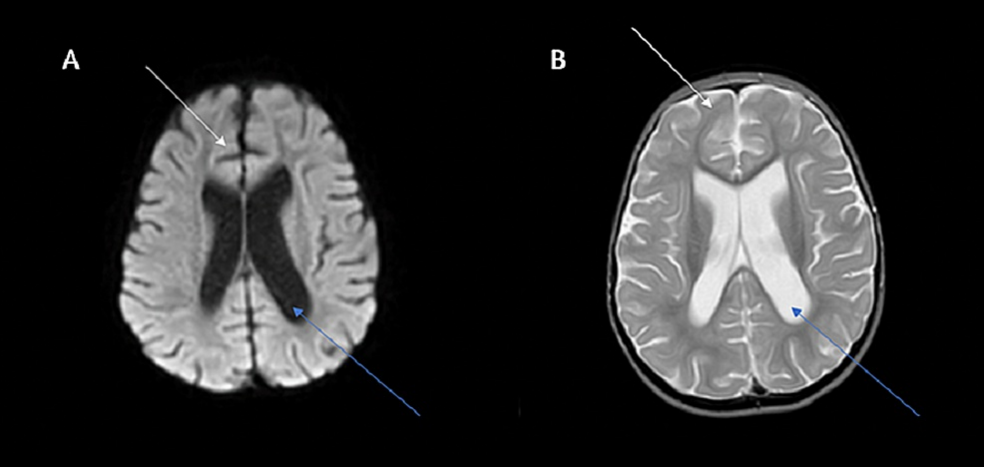
Image A: T1 MRI sequence, axial view Image B: T2 MRI sequence, axial view: both images show ex vacuo ventriculomegaly (blue arrows) and frontal atrophy (white arrows). MRI: magnetic resonance imaging

There was poor differentiation of the gray-white matter interface in the temporal and frontal lobes, with atrophy in the frontal lobe and infratentorial region. Secondary ex-vacuo ventriculomegaly was also seen. Figure 1 shows the MRI findings in this patient.

**Figure 2:**
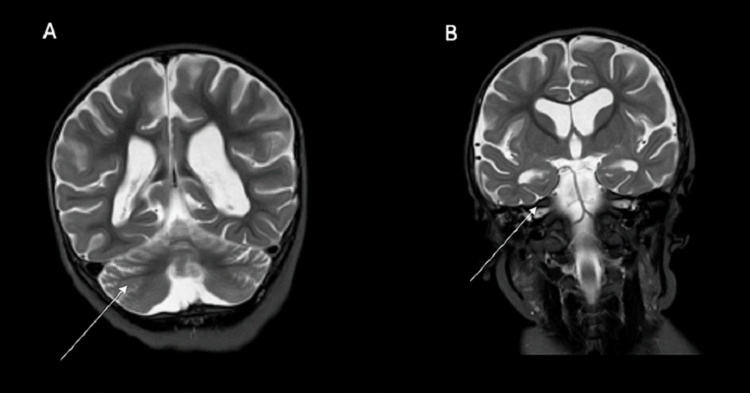
Panel A: T2 MRI sequence, coronal view; Panel B: T2 MRI sequence, coronal view Both images show cerebellar atrophy (white arrows).

Figure 2 shows cerebellar atrophy in the patient.

Electroencephalogram (EEG)

At times, there was high amplitude with irregular spikes and waves. Then, the spikes and waves were immediately followed by brief diffuse attenuation +/- overlying fast activity, often without a clear clinical correlation but at times with an associated myoclonic jerk. At least eight of these seizures were noted within 24 hours.

Impression: The EEG was markedly abnormal when awake and asleep. There were abundant multi-focal and diffuse epileptiform discharges and multiple recorded seizures with multifocal onset. Findings were consistent with epileptic encephalopathy and a predominance of myoclonic or brief tonic seizures.

Genetic testing

The first mutation was already reported, and an additional test shows an additional deletion of unknown significance.

1) MYO5A, located at band 15q21, classical of GS.

2) Loss 18p11.32p11.31 (2.1 MB): Previously reported in a patient with refractory seizures and developmental delay [6].

Impression and treatment: The ketogenic diet was weaned due to the lack of efficacy. He was discharged with the following medications: lacosamide 40 mg BID, clobazam 10 mg TID, levetiracetam 500 mg BID, cannabinol 500 mg, zonisamide 50 mg, and diazepam PRN.

The prognosis of the patient is mainly unknown due to the natural course of the disease. The patient also had an additional mutation [loss 18p11.32p11.31 (2.1 MB)], which could be worsening the condition.

## Discussion

Chédiak-Higashi syndrome, Elejalde ­­syndrome, Hermansky-Pudlak syndrome type 2, and GS share similar features. All the syndromes present with partial albinism. Neurological features can be seen in all the differentials, except GS3. Immunodeficiency is seen in all the disorders except in Elejalde syndrome and GS1.

Making the differential diagnosis of these three syndromes could be challenging. Table 2 showed the main differences among these three disorders [4,7-9].

**Table 2:** Diffrential dianosis of Griscelli syndrome MYO5A: Myosin-Va; RAB27A: Ras-related protein Rab-27A

Condition	Clinical Manifestations	Genetics	Epidemiology
Chédiak–Higashi syndrome [8]	Partial albinism. Retina pigmentation, impaired visual acuity, photophobia, increase red reflex, nystagmus. Early-onset immunodeficiency. Mucosal bleeding, easy bruising. Neurologic manifestations appeared in early adulthood. They included: ataxia, tremors, motor, and sensory neuropathies, absent deep tendon reflexes. Accelerated phase: fever, hepatosplenomegaly, lymphadenopathy, neutropenia, anemia, thrombocytopenia, most patients die within the first ten years of life. Patients have an increased risk of developing a stroke.	Autosomal recessive. Lysosomal trafficking regulator is located in chromosome 1 (1q2-q44).	Unknown exact prevalence. Fewer than 500 cases worldwide.
Elejalde ­­syndrome [7]	Partial albinism, not immunocompromised early-onset neurologic dysfunction: marked hypotonia, hyporeflexia or hyperreflexia, spastic or flaccid hemiplegia or quadriplegia, seizures, ataxia, developmental delay. Ophthalmologic manifestations included: nystagmus, diplopia, pupilar areflexia, congenital amaurosis.	Autosomal recessive. MYO5A gene mutations.	20 cases have been reported up to 2019.
Hermansky-Pudlak syndrome type 2 [9]	Partial albinism, neutropenia, low platelet count, microcephaly, horizontal nystagmus, mental retardation.	Autosomal recessive disease. Mutations in AP3B1 gene on chromosome 5. The protein function is lysosomal trafficking.	Unknown
Griscelli Type 1 [4]	Partial albinism, not immunocompromised, neurological deficits, severe developmental delay, and mental retardation.	Mutation in the MYO5A gene on chromosome 15q21.	Only 150 cases have been reported.
Griscelli Type 2 [4]	Partial albinism, immunodeficiency, hemophagocytic syndrome. The hemophagocytic syndrome cause infiltration in the organs, including the brain.	Mutation in the RAB27A gene on chromosome 15q21.	
Griscelli Type 3 [4]	Partial albinism. without neurological or immunological compromise. Only minor features are seen in GS3.	Mutation in either melanophilin or MYO5A genes.	

Additionally, we looked at other reported cases of GS and seizures in pediatric populations. We used an advanced PubMed strategy with the following terms: (Griscelli syndrome[Title/Abstract]) AND (seizures[Title/Abstract]). We found 12 cases with both features. Table 3 shows the reported GS cases, either type 1 or 2, with seizures [1,4,10-18].

**Table 3:** Reported cases of Griscelli syndrome with seizures

Author, year	Type of GS, age, sex	Clinical Features	1) Age of onset, 2) treatment, 3) Outcome.	MRI findings
Russ et al 2019 [11]	Griscelli Type 2, 1 year, female.	Status epilepticus, encephalitis, oculocutaneous albinism, pancytopenia, hypertriglyceridemia, developed hemophagocytic lymph histiocytosis at year one.	Three months. Status epilepticus related to Influenza. She was treated with levetiracetam, oxcarbazepine, and fosphenytoin. A year later, she continued to have refractory epilepsy.	Hyperintensities of the corona radiata and periventricular white matter.
Masri et al, 2008 [1]	Griscelli type 2, 6 years, male	Developmental delay, silver-gray hair, bronze color skin, generalized hypotonia, anemia.	Six years old. No treatment discuss. Focal seizures related to a fever of unknown origin. Two weeks later, he develops generalized tonic-clonic seizures; The patient died because of rapid neurological deterioration.	MRI showed white and grey matter involvement and periventricular hyperintensities.
Saini et al, 2014 [12]	Griscelli type 2, 3 years, male.	Silvery-gray hair, spastic quadriparesis, hyperreflexia, hepatosplenomegaly, abnormally pigmented skin.	Three years old. Not treatment discuss. Focal seizures related to a viral process that caused an altered level of consciousness.	Hyperintensities in the cerebellar hemisphere and splenium of the corpus callosum.
Cagdas et al, 2012 [13]	Griscelli type 1, 7 months, male	Silver-gray hair, developmental delay, hypotonia, lack of head control	Seven months. Currently, at age four, his seizures are well-controlled with carbamazepine and clonazepam. He was experiencing seizures for four months. EEG showed frequent sharp waves in the left hemisphere. Seizures were difficult to control.	Normal MRI reported at 1.5 years.
Sahu et al, 2017 [14]	Griscelli type 2, 11 months, male.	Silver-grey hair, increased tone, pancytopenia	Convulsion since he had 18 days of life. No treatment discussed. The patient died two months after the onset of symptoms	Multiple hyperdense foci in the white matter of the left hemisphere; Cortical and subcortical hyperintense lesions on MRI.
Ashrafi et al, 2006 [10]	Griscelli type 2, 6 years, male	Silvery-gray hair, decreased level of consciousness, seizure disorder, fever.	Six years old. No treatment discussed. He had seizures and subsequent stupor with tonic-clonic seizures. The patient died 12 days after admission, due to an accelerated phase of hemophagocytic syndrome.	There are frontal, cortical, and subcortical lesions; white matter lesions in the periventricular areas. Also, there is hyperintensity in the upper pons, quadrigeminal plate, upper pons, bilateral caudate, lentiform nucleus, and splenium of the corpus callosum.
Panigrani et al. 2015 [15]	Griscelli type 2, 1 year, female	Silvery-gray hair, hepatosplenomegaly, normal WBC.	One year old. She was treated with phenytoin, levetiracetam, valproate, midazolam infusion. Status epilepticus remained refractory until receiving ketamine. Left frontal seizures and status epilepticus. It was very difficult to stop the convulsions. She continued to have intractable seizures and died on day 14 of hospitalization.	On T2/Flair: No hyperintensities in the periventricular area, cerebellar hemispheres, and white matter.
Alva-Moncayo et al, 2003 [16]	Griscelli, 16 months, male, most probably Griscelli type 2	Developmental delay, silver-grey hair, pancytopenia, and hepatosplenomegaly.	16 months of age. No treatment discussed. Generalized, tonic and refractory seizures that progressed to a vegetative state.	MRI showed diffuse white matter demyelination, mainly in the bilateral frontotemporal area; a biopsy of the skin showed hyperpigmentation with accumulations of melanin.
Dinakar et al, 2003 [17]	Griscelli, 6 years, female, most probably Griscelli type 2	Anasarca, jaundice, splenomegaly, silver-grey hair, horizontal nystagmus, retinal pigmentation, generalized hypertonia, hyperreflexia, and pancytopenia. Her IQ is 67. She also has a regression of milestones since age four.	Seizures started at age two. No treatment discussed.	CT scan showed a Dandy-Walker cyst.
Thomas et al, 2009 [4]	Griscelli type 1, 2 years, male	Silver-grey hair, hypotonia, developmental delay.	Two years old. No treatment discussed. Multiple seizures, despite multiple medications. The seizures remained difficult to manage.	Small atrophic cerebellum.
	Griscelli type 1, 2 months, female	Silver grey hair, hypotonia, motor delay.	Two months old. Treatment not discussed. EEG showed a hypsarrhythmia pattern.	Information not available.
Elmaksoud et al, 2020 [18]	Griscelli type 1, 7 months, male	Silvery-grey hair and eyebrows, developmental delay, hypotonia.	Seven months old. No treatment discussed. Seizures control with multiple medications. The other sibling has GS1 but did not have any seizure.	Bilateral symmetrical widening of the Sylvian fissure.

On literature search, we found four cases of GS1 with seizures and eight cases of GS2 and seizures. Two out of four cases seem to have refractory epilepsy while the others only have mild epilepsy [4,18]. Interestingly, in one report, two siblings had the same mutation, and both were diagnosed with GS1, but just one sibling had epilepsy, showing that different phenotypes could be present with the same mutation [18]. Five out of eight cases of GS2 died shortly after developing seizures. One patient developed seizures and progressed to a vegetative state [16]. Another patient continued to have refractory epilepsy a year later; the first episode. There is no description of what medication was tried [11]. Our patient has refractory epilepsy, and he was treated with more medications than other previously described cases.

There have only been 20 cases reported of GS1 based on our literature review, so information is limited. The most common neurological manifestations are hypotonia, seizures, neurodevelopmental delay, and ophthalmological features [4]. In our case, the patient did not have ocular manifestations. Once the diagnosis of GS1 has been made, it is important to differentiate it from GS2, which can also cause neurological symptoms. Congenital cerebellar atrophy is the most common MRI sign in GS1 [10]. The main findings in our patient were cortical atrophy, cerebellar atrophy, and ex-vacuo ventriculomegaly.

The neurological features of GS1 are related to the gene MYO5A, which regulates the organelle transport in melanocytes and neuronal cells [5]. The abnormal movement of melanosomes altered intracellular vesicle transport, so fast axonal transport in nerve cells is reduced. Neuronal development, axonal transport, dendritic spine structure, and synaptic plasticity altered [19]. The neurological features of GS2 are related to the accelerated phase of HPS due to histiocytic infiltration in the central nervous system [10]. The gene regulates the docking of proteins and exocytosis of granules containing granzyme and perforins. The lack of granzymes and perforins explained the immunodeficiency in GS2 [4]. Neurological manifestations in GS2 can be a sign of an accelerated phase of HPS [10].

Microscopic examination of the hair shafts allows differentiating GS from similar disorders like Chediak-Higashi and Elejaldes syndrome [1]. In GS, there are large clumps of melanin distributed irregularly. In Chediak-Higashi, there are small clumps of melanin regularly distributed, and in Elejaldes syndrome, the clumps of melanin are distributed very similarly to GS [1]. Overall the prognosis is poor, and the limited information about GS1 left physicians without many options. Currently, bone marrow transplantation seems to be the only definitive treatment for GS [15]. Cannabinol was the anti-seizure medication more effective in the patient. There are no studies about GS and cannabinol. However, in other epilepsy syndromes of childhood, such as Dravet's syndrome and Lennox Gastau, cannabinol reduced the frequency of seizures by 37.2% in Lennox Gastau and 38.9 in Dravet's syndrome [20].

Interestingly, our patient also had a deletion on 18p11.32p11.31. This finding is remarkable because there is a case report by Verroti et al. describing a case with a deletion in 18p11.32p11.31. This patient presented with similar clinical features as our patient, including drug-resistant atypical absence epilepsy and severe developmental delay [6]. One crucial gene found in this region is the TGIF-1, expressed very early in the nervous system. Mutation in the TGIF-1 has been related to severe mental retardation and holoprosencephaly [6]. While seizures are expected in GS1. The frequency and severity of the seizures in our patient are more severe than other cases of GS1 with seizures. We believe that mutation in the MYOV5A gene combined with the deletion 18p11.32p11.31 in our patient explained the severity of the case.

## Conclusions

We found four cases of GS1 with seizures and eight cases with GS2 and seizures. The neurological manifestation is GS1 is related to the MYO5a while the GS2 neurological manifestations are indirectly related to the HPS when there is an accelerated phase. The differential diagnosis between Chédiak-Higashi syndrome, Elejalde ­­syndrome, and GS could be challenging. The microscopic finding is the most accurate method to differentiate among these disorders. The prognosis of these patients is poor, the same as in our patient. The mutation in the MYO5A gene and the 18p11.32p11.31 deletion could explain the severity of the seizures in our case. This is the first report of GS1 with a deletion in 18p11.32p11.31.
